# Efficient delivery of DNA into bovine preimplantation embryos by multiwall carbon nanotubes

**DOI:** 10.1038/srep33588

**Published:** 2016-09-19

**Authors:** Michele Munk, Luiz O. Ladeira, Bruno C. Carvalho, Luiz S. A. Camargo, Nádia R. B. Raposo, Raquel V. Serapião, Carolina C. R. Quintão, Saulo R. Silva, Jaqueline S. Soares, Ado Jorio, Humberto M. Brandão

**Affiliations:** 1 Department of Biology, Federal University of Juiz de Fora, 36036-900, Juiz de Fora, Brazil; 2 Department of Physics, Federal University of Minas Gerais, 31270-901 Belo Horizonte, Brazil; 3 Brazilian Agricultural Research Corporation, Embrapa Dairy Cattle (CNPGL), 36038-330 Juiz de Fora, Brazil; 4 Center of Research and Innovation in Health Sciences (NUPICS), Federal University of Juiz de Fora, 36036-900 Juiz de Fora, Brazil; 5 Department of Physics, Federal University of Ouro Preto, 35400-000 Ouro Preto, Brazil

## Abstract

The pellucid zone (PZ) is a protective embryonic cells barrier against chemical, physical or biological substances. This put, usual transfection methods are not efficient for mammal oocytes and embryos as they are exclusively for somatic cells. Carbon nanotubes have emerged as a new method for gene delivery, and they can be an alternative for embryos transfection, however its ability to cross the PZ and mediated gene transfer is unknown. Our data confirm that multiwall carbon nanotubes (MWNTs) can cross the PZ and delivery of pDNA into *in vitro*-fertilized bovine embryos. The degeneration rate and the expression of genes associated to cell viability were not affected in embryos exposed to MWNTs. Those embryos, however, had lower cell number and higher apoptotic cell index, but this did not impair the embryonic development. This study shows the potential utility of the MWNT for the development of new method for delivery of DNA into bovine embryos.

Introduction of foreign genes into target cells is a crucial step for achievement of gene therapy^1^, study of gene function and human diseases^2^ and production of human proteins for the pharmaceutical in biofactories^3^. Various methods for somatic cell transfection have been described including electroporation^4^, cationic polymers^5^, liposomes^6^ and virus-mediated method^7^. In embryonic cells, the pellucid zone (PZ) is a major obstacle to widespread use of these available methods. The PZ is an important biological barrier that protects the mammalian embryo during the pre-implantation stage inhibiting the contamination with chemical, physical or biological substances (*e.g.* virus)^8^,^9^ capable of injuring the embryonic stem cells that give rise to fetus. For this purpose, the most widespread methods to cross the PZ and introducing nucleic acids are based on injection of lentiviral vectors into perivitelline space of fertilized one-cell eggs^10^, microinjection of the desired gene into zygote pronucleus^11^,^12^, or injection of spermatozoa carrying exogenous DNA^13^. However, in these systems there are limitations such as requiring complicated procedures, expensive equipment, and excessive manipulation of the embryo resulting in low viability^14^. Thus, the current gene delivery systems constantly needs to progress, increasing efficiency and reducing cost. This has brought about the necessity to develop alternative strategies that solve these problems.

Recently, among various nonviral gene vectors, nanomaterials offer an ideal platform for the incorporation of all the desirable characteristics into a single gene delivery system^15^. In somatic cell types, carbon nanotubes (CNT) were used for transport of therapeutic molecules or genes^16^,^17^. In these cells, CNT can cross the cellular membrane through two mechanisms: endocytosis^18^ or an energy-independent nonendocytotic mechanism that involves the insertion and diffusion of CNT through the lipid bilayer membrane^19^. Therefore, the relentless search for alternative nanomaterials for gene delivery has led to the utilization of CNT as a viable candidate.

Few experiments using mammalian embryos and CNT have been conducted, and such studies used embryonic stage PZ-free and fetus^20^,^21^. These studies were designed and conducted to characterize and evaluate the toxicological potential of CNT in laboratory animals, however, these researches did not involve biotechnology applications, such as gene delivery. The limitation of using transfection methods traditionally employed in somatic cells for embryos transfection is the presence of the PZ. To be an alternative for gene delivery to pre-implantation embryo stage, the CNT should cross PZ without impairment of embryo viability. Other issue is the question of achieving large-scale transfection of embryos. So far there are no reports of delivery of DNA into mammalian embryos without their individual handling.

The bovine embryo is a well-known animal model, which has been extensively studied. The increasing interest in the bovine embryo as a model in biological research is related to its similarity with a human embryo^22^, simplicity, a large number of oocyte can be easily obtained from a slaughterhouse and at low cost. Moreover, among all transgenic mammalian bioreactors already produced, bovines are able to produce the largest volume of milk, making easy to obtain high amount of recombinant proteins^23^. Thus, the aim of the present study was to determine whether multiwall carbon nanotubes (MWNTs) can pass through the PZ; delivery plasmid DNA into *in vitro*-produced bovine embryos; and its effects on mammalian pre-implantation embryonic development.

## Results and Discussion

**Figure 1:**
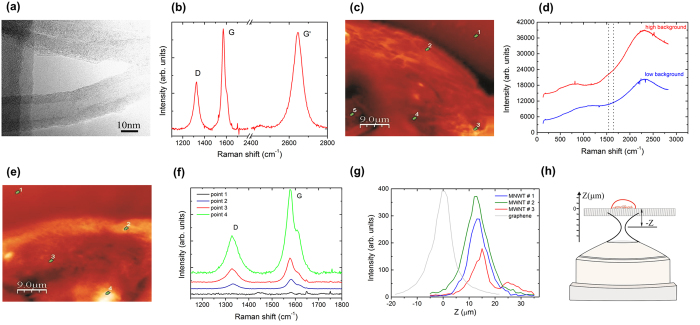
Transmission electron microscopy and Raman imaging and spectroscopy of MWNTs and bovine blastocysts exposed to MWNTs (0.2 μg ml^−1^). (**a**) Shows the TEM of a MWNT with diameter of approximately 20 nm; **(b)** shows the Raman spectrum of the carbon nanotubes, evidencing the three spectral signatures: D band at 1350 cm^−1^, G band at 1580 cm^−1^ and D band at 2700 cm^−1^; (**c**) shows the optical imaging of an unexposed embryo, which is dominated by embryo luminescence (see panel (**d**); the four different points (1–4) depicted in **(c**) indicated the outside of the embryo (point 1); the interface between the PZ and air (point 2); the PZ (point 3); and the inside of the embryo (point 4). **(d)** Shows the typical luminescence signal from the control group; **(e)** Raman imaging of a MWNT-exposed embryo with an optical filter to select the scattering from the Raman G-band (1550–1650 cm^−1^); **(f)** Raman spectra at different points (1–4) depicted in (**e**): point 1, outside of the embryo; point 2, interface between the PZ and air; point 3, PZ; and point 4, inside of the embryo; Notice the stronger Raman signal from inside the embryo. (**g**) Z-scan confocal G band Raman imaging of three locations inside the embryo (MWNT #1, #2 and #3), where the MWNT G band signal was strong; The gray profile in (**g**) is the G-band intensity Z-scan for a controlled, one-atom-thick graphene sheet deposited on a quartz substrate, which provided the reference for the Z = 0 in the Z-axis, and the spatial resolution of the confocal Raman system (ΔZ = 7.8 μm). **(h)** Schematic showing the Z-scan experiment.

From the Figure Supplementary 1 it can be observed typical morphologies of the MWNT and diameters that ranged from 20 to 40 nm and lengths that ranged from 40 to 60 μm. The MWNTs were produced by floating catalytic chemical vapor deposition^24^ and the purity of the MWNT sample we used was 81% (wt. %) (Supplementary Fig. 2). Figure 1a shows a transmission electron microscopy (TEM) image of the MWNTs used in this study, which were evaluated by TEM and Raman spectroscopy (Fig. 1b). The Raman signatures of the MWNTs appear as peaks located at 1350 cm^−1^, 1580 cm^−1^ and 2700 cm^−1^, as shown in Fig. 1b. These Raman features are common to all sp^2^ carbon forms^25^. The feature at 1580 cm^−1^ is named G band (G from graphene, the mother material of DWNTs), and it is the signature of the C-C stretching vibrational mode. The feature at 1350 cm^−1^ is named D band, and it is the Raman signature of the six C atoms in the hexagonal benzene-like structure breathing. This peak can only be observed in the Raman spectrum of DWNTs where there are defects in the tube walls (here the reason for the name D band), which are introduced either in the growth or in the functionalization processes. The feature at 2700 cm^−1^ is a second-order of the D band, appearing at twice the D band frequency, although it does not require defects to be active. This band is called G′, and those Raman signature labels are all depicted in Fig. 1b.

Figure 1c shows an image of the embryo before its exposure to the MWNT aqueous solution. This image is made by acquiring the intensity of scattered light along the sample. The yellowish areas indicate the embryo, which appears in the imaging owing to an increase in the spectroscopic background, as shown in Fig. 1d. The two vertical doted lines enclose the spectral range where the Raman G band should appear when a DWNT is present, but it is absent here since this imaged embryo was not exposed to the MWNT aqueous solution. The points 1–4 are the embryo’s outer side, air/PZ interface, PZ and the inside of the embryo, respectively. In these four points, the characteristic MWNT Raman peaks were not observed.

To study CNT embryo permeation, we exposed bovine embryos to 0.2 μg ml^−1^ MWNTs for 24 h. The selection of this concentration was based on previous studies that evaluated the biocompatibility of carboxylated MWNTs on bovine embryos^26^. Our results demonstrated that the MWNT were able to pass through the PZ. Figure 1e shows a Raman spectroscopy image of a MWNT-exposed bovine embryo. In this case, the G-band (with a baseline adjustment of the Raman spectrum) was not observed at point 1 (embryo outer side), which indicates the absence of the MWNTs outside the embryo (black-line spectrum in Fig. 1f). At the air/PZ interface (point 2), it was possible to detect a slight stretch of the G- and D-bands (blue-line spectrum in Fig. 1f), showing the presence of MWNTs even after washing the air/PZ interface. In the PZ (point 3), there was an increase in the intensity of both Raman bands (red-line spectrum in Fig. 1f), which reached a maximum intensity in some specific locations inside the embryo (e.g. point 4 in Fig. 1e, and respective green-line spectrum in Fig. 1f). These findings indicate the presence of MWNTs inside the embryo.

In order to check the localization of MWNT inside the embryo we performed a Z-scan confocal Raman experiment of the G-band intensity, as depicted in Fig. 1g. Figure 1h shows a schematic of this Z-scan experiment, where Z represents the distance of the laser focal point that was referenced to the embryo-substrate interface (Z = 0). Since the Raman signal in the confocal system comes only from the focal region, by scanning the focus along Z we are able to provide the location of the spectral information in the Z direction, i.e. whether the Raman emitter is below the substrate-cell interface (Z negative), at the interface (Z = 0) or inside the cell (Z positive). We performed three Z-scan profiles at three locations where the spectral response from MWNTs were strong inside the embryo, similar to point 4 in Fig. 1e (named MWNT #1, #2 and #3). For the locations MWNT #1 and #2, the strongest G-band intensities were found to be at 13.5 μm (blue trace in Fig. 1g) and 12.7 μm (green trace in Fig. 1g) above the embryo-substrate interface (Z = 0) in the Z axis. Besides being located inside the embryo (Z positive), they were also observed to be distributed inside, since both full widths at half maximum (FWHMs, of 8.1 and 10.2 μm, respectively) were slightly larger than Z-scam spectral resolution ΔZ (see caption to Fig. 1). At the third location (MWNT #3), the G-band spectral profile was found to be even more broadly distributed along Z, with two peaks at 15.1 and 25.5 μm (see red trace in Fig. 1g). These results prove the MWNT material is indeed located inside the cell in both three dimensions (X, Y and Z), and they are distributed along different highs inside the cell, along the Z direction.

**Figure 2:**
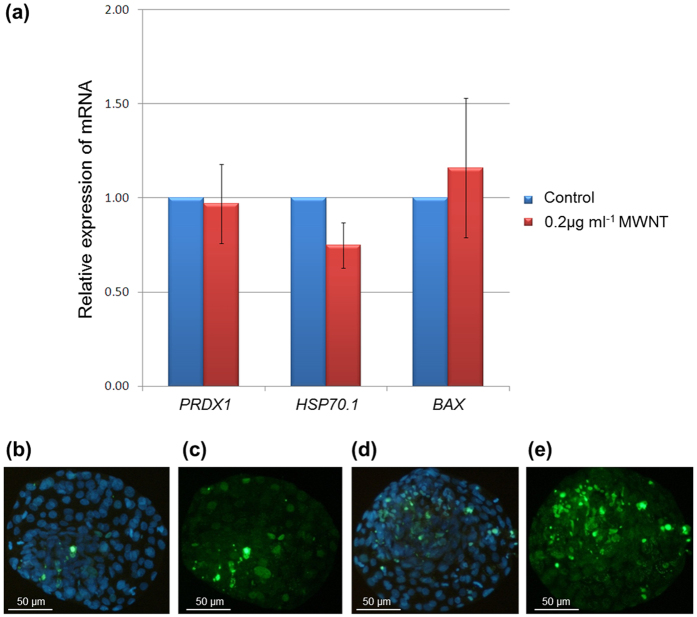
Representative images of TUNEL-labeled nuclei and the relative expression of genes in bovine blastocysts cultured with MWNTs (0.2 μg ml^−1^) for 72 h. (a) Shows the expression of PRDX1, HSP70.1 and BAX genes in bovine blastocysts cultured without MWNTs (control group) and with MWNTs (0.2μg ml-1). MWNTs (0.2μg ml-1) compared with the control group (relative expression=1.00) (p>0.05; mean±S.E.M.). (**b**,**c**) Show the total number of cells (blue fluorescence) and the number of apoptotic cells (green fluorescence), respectively, from blastocysts of the control group; (**d**,**e**) Show the total cell number and apoptotic cells, respectively, from blastocysts of cells cultured with MWNTs (optical microscopy fluorescence with 100x magnification).

After achieving these results, which demonstrated that MWNT can cross the PZ of mammalian embryos touching the embryonic cells, we studied the effects of MWNTs inside the embryos, in the same MWNT concentration. We evaluated the rates of embryonic development, embryo hatching, embryo degeneration and *in situ* apoptosis. There was no difference (p > 0.05) in the hatching rate and degeneration at 24, 48 or 72 h of culture between the control and the MWNT-exposed embryos (Table 1). However, blastocysts exposed to the MWNTs had lower (p < 0.05) total cell numbers and a higher (P < 0.05) apoptotic cell index compared to blastocysts from the control group (Table 2; Fig. 2b–e), although the number of apoptotic cells was similar (p > 0.05) in the both groups (Table 2). These results are in agreement with previous studies that showed that the exposure of hamster lung cells^27^ and mouse embryonic stem cells^28^ to MWNT can induce apoptosis by DNA damage. The mechanisms by which CNTs can cause toxicity to cell are not fully understood, but the toxic effects may occur for two reasons. First, chemical reactions can result from an inflammatory response to the CNTs; for example, an oxidative stress response might be stimulated by the size of the CNTs or by the presence of impurities (such as metals, amorphous carbon or silica) in the CNT samples^29^. Second, toxic effects might be caused by the physical action of CNTs, which have a needle-like shape that can damage the cell membrane and interfere with compartmentalization in the cell^30^. Thus, the ratio of length to width of the CNTs and the purity of the CNTs can be determinants of the toxicity of CNTs^29^,^30^,^31^.

In normally developing embryo, both *in vivo* and *in vitro,* some cells spontaneously undergo apoptosis^32^,^33^. It has been shown that *in vitro* cultured mammalian embryos showed a higher level of apoptosis compared to their *in vivo* counterparts^34^. Apoptosis is a physiological process of cell elimination that protects the organism maintaining homeostasis^35^ and it is necessary for embryonic development^36^,^37^. Although, the high apoptotic cell index is associated with a lower embryo quality^38^ it is possible that the apoptosis eliminating potentially non-viable cell embryo exposed to MWNT without necessarily compromising the potential for further development.

Makarevich *et al*.^38^ found that after removing the embryo from the stress conditions, the embryos with high apoptotic cell index are able to regenerate, which were proved by the increase in embryo cell number and normalizing apoptotic cell index. In addition, there are strong evidences that cells incubated with CNTs in culture can lead to cycles of initial senescence with subsequent proliferation after a certain critical concentration is reached^39^. In the present study, others parameters of embryo viability as degeneration and hatching rates were maintained. Thus, apoptosis index is a predictor of cytotoxic mechanisms, but the others molecular biomarkers of embryo quality should be evaluated.

To check that the embryonic viability, we also examined the expression of genes associated with apoptosis (*BAX*, which stands for BCL2-associated X protein) and response to stress (*HSP70.1* - heat shock 70 kDa protein 1A; *PRDX1* – peroxiredoxin) by Real Time PCR analysis following the MWNTs treatment. The relative amounts of *BAX* (1.16 ± 0.37), *HSP70.1* (0.75 ± 0.12) and *PRDX1* (0.97 ± 0.21) transcripts in embryos cultured with MWNTs were not different from those in the control group (p > 0.05) (Fig. 2a). The effect of CNT on the gene expression of somatic cells in the literature is conflicting. Roman *et al*.^40^ found that single-walled carbon nanotubes (SWNT) can disturb the expression of cell proliferation, apoptosis, survival and angiogenesis genes in the tissues from avian embryo whereas Hirano *et al*.^41^ reported that the exposure of mouse macrophages to MWNTs increased the expression of only some interleukin genes, despite its cytotoxic effect. Pereira *et al*.^42^ reported that 100 μg mL^−1^ cellulose nanofibers (CNF), a needle-like fibers similar to CNT, did not alter the gene expression of HSP70.1, and PRDX1 genes in bovine fibroblast *in vitro* cultured.

Hirano *et al*.^41^ evaluated macrophages that constantly generate reactive oxygen species as a part of their normal cellular reactivity, unlike the present study, which has studied the influence of the MWNT in embryo preimplantation stages. The toxicity observed by Roman *et al*.^40^ may be related to the biological model (embryo/fetus avian), concentration (approximated values of 0.6 μg ml^−1^ per egg), CNT type (SWNT) and exposure time (12 days). In the present study, mammalian embryos were *in vitro* exposed to 0.2 μg ml^−1^ MWNT for 72 h. In addition, Roman *et al*.^40^ used toxic concentration previous tested, while we used concentrations biocompatible with bovine embryos accordingly to our previous toxicological studies with MWNT^26^. In fact, our findings of degeneration and hatching rate and gene expression corroborates this potential application of MWNT. Furthermore, data from gene expression is according to our previous studies using other fibrous nanomaterial in bovine fibroblast^42^ which showed that low concentration of CNF does not induce cytotoxicity. In the present study, we report the gene expression levels in preimplantation bovine embryos and the results show that MWNT not interfered in biomarkers genes related to cell stress response, oxidative stress and to apoptosis control.

Regarding the length of CNT, some studies have shown that MWNT with longer length have greater toxicity than smaller ones^43^. However, others studies contradict these findings by demonstrating that the shorter MWNT caused more toxicity than longer-length MWNT^44^. Despite the potential advantages of using MWNT in gene delivery, little is known about the effects of these NMs on mammalian embryos. Few tests about toxicity of CNT in embryos have been performed on mammalian species embryos. Lim *et al*.^45^ showed that maternal exposure to MWNT (length 20 μm) does not induce embryo-fetal developmental toxicity in rats. However, another study reported development abnormalities in mice exposed to MWNT (length 2 μm)^46^. In the present study, a prolonged exposure time (72 h) to MWNT (length 40–60 μm) induced apoptosis, but did not affect hatching and degeneration rates. For the gene delivery applications, embryos were exposed for 12 h to MWNT only. It is possible to hypothesized that, regardless the size a less exposure time may decrease the effects of MWNT in the apoptotic index. Here, we assumed that if the embryos remained viable after MWNTs exposition they could be used for a new biotechnological application, such as gene delivery to embryo, solving thus the limitations of gene delivery techniques that require embryo manipulation.

Therefore, we have studied the potential utility of MWNT as an alternative method to transfection of bovine embryos. The binding of pDNA to MWNT was investigated by Raman spectroscopy. pDNA interaction with MWNT causes to increase of the vibrations in the 1608 and 1279 cm^−1^ (Supplementary Fig. 3). According to previous studies^47^ the intensification and shift absorption of phosphate band at 1095 cm^−1^ has been found in pDNA-MWNT complex (Supplementary Fig. 3). Previous studies showed that this interaction of the DNA with CNT is based on wrapping of nucleic acid molecules around CNT^47^,^48^,^49^. These findings demonstrate the interaction of pDNA with MWNT.

**Figure 3:**
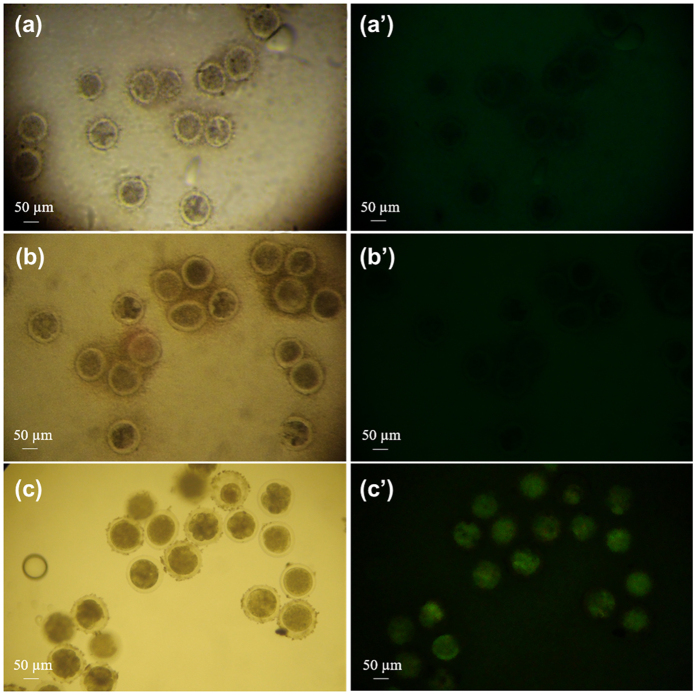
GFP expression of transgenic embryos (day 3). (**a**,**a**’) Control embryo (GFP-); (**b**,**b**’) Plasmid alone (GFP−). Transgenic embryo (GFP+). Images under visible light (**a,b**) and fluorescent light (**a**’,**b**’). Optical microscopy fluorescence with 100x magnification.

MWNT were complexed directly with the GFP plasmid, which encodes the green fluorescent protein (GFP) to form MWNT-GFP carries and used to transfect *in vitro*-fertilized bovine zygotes. GFP expression was evaluated as *in-situ* fluorescence at 3 days post-fertilization (2 to 8-cell stage) (Fig. 3). Fluorescence was not observed with control embryo incubated with pDNA only (Fig. 3b). The findings of the current study are consistent with those Carballada *et al*.^50^ who observed no free diffusion of plasmid into the embryos cells. It has been shown in previous studies that PZ prevents transfection of mammalian embryo *in vitro*^51^,^52^,^53^. This probably occurs because the PZ (10.5-μm thick)^8^ acts as a barrier that hinders the free diffusion of GFP-plasmid between embryo cell cytoplasm and external medium. Because of that, the current transfection methods require drilling or removal of PZ^50^,^51^,^52^,^53^. Fluorescence analysis showed that when MWNTs were used, they formed complexes with the pDNA and these complexes were subsequently permeated to the embryo. Then, pDNA was able to reach the nucleus, allowing transcription and translation of the GFP (Fig. 3c). Raman spectroscopy data from this study showed that pDNA can interact with MWNT (Supplementary Fig. 3). DNA molecules may be encapsulated inside or wrap around MWNT owing to van der Waals attraction and electrostatic interactions between DNA and MWNT^54^,^55^. Non-covalent binding of DNA to the surface of the CNT favors their protection from degradation by cytoplasmic nucleases^56^ increases the efficiency in releasing the contents into the cell nucleus^57^. The compact structure of the GFP-plasmid/MWNT resulting from condensation should facilitate embryo cytoplasm penetration, thereby enhancing the pDNA delivery efficiency. In addition, transfected embryos were fully capable of development *in vitro* till the blastocyst stage. One of the most significant aspects of utilization of MWNT for gene delivery in mammalian embryos is the simplicity of this new method for gene delivery. The insertion of the pDNA by microinjection or vector viral are methods that require complicated procedures and few embryos are transfected per day. However, using the pDNA-MWNT complex the only limitation is the number of embryos available for the procedure. With this technique is possible to transfect various embryos simultaneously.

**Figure 4:**
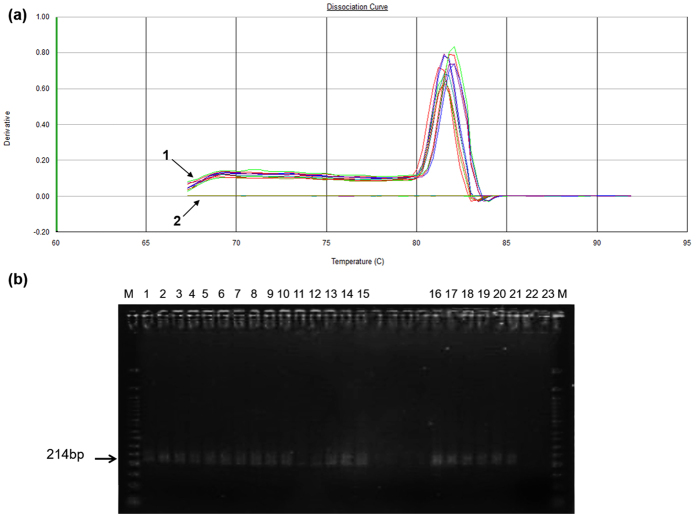
Analysis of the presence of GFP transgene in transfected embryos. (**a**) Real time PCR detection of GFP sequences in the transfected embryos. 1. The melting curve for reactions each characterizing the expression of GFP gene. The curve contains only one peak indicating that the reaction generates only one product. 2. Non-tranfected embryo or control without template. (**b**) Gel agarose analysis of the presence of GFP transgene transfected embryo. PCR products were fractionated in 2% agarose gel. Screening for the transgene was performed by PCR encompassing 214 bp. “M” shows the molecular weight markers (50 bp). Lanes 1–15 contains the product from the PCR using as template transfected embryos with pDNA-MWNT (1:20 relation). Lanes 16–18 contains the product from the PCR using as template transfected embryos with pDNA-MWNT (1:10 relation). Lanes 19–21 contains the product from the PCR using as template transfected embryos with pDNA-MWNT (1:1 relation), Lane 22 represent non-tranfected embryo, Lane 23 represents a control without template.

Real time PCR examination was performed to ensure the presence of GFP in embryos. A melting curve was used to check the specificity of the amplified product. No non-specific products or primer dimers appeared in PCR products after melting curve (Fig. 4a). The band of PCR fragment of GFP gene was also detected by electrophoresis analysis, confirming the presence of the transgene in the transfected embryos (Fig. 4b). PCR amplification was checked on 2% agarose gel. Lanes 1–15 represent embryos transfected with relation 1:20 of pDNA-MWNT, Lanes 16–18 represent embryos transfected with relation 1:10 of pDNA-MWNT and Lanes 19–21 represent embryos transfected with relation 1:1 of pDNA-MWNT (Fig. 4b). All analyses resulted in the location of these unique bands in the gel. Fragments of 214 pb were detected confirming the specificity of the PCR reaction. To optimize the proportion of pDNA to MWNT, we made a series of pDNA-MWNT mixtures at different mass ratios: 1:1, 1:10 and 1:20. From PCR analysis, the percentage of GFP-positive embryos were 3/30 (10%) for relation 1:1 and 1:10 pDNA-MWNT and 15/30 (50%) for relation 1:20 of pDNA-MWNT. The rate of GFP in 1:1 and 1:10 was considerable low compared with 1:20. It is possible that these embryos were not effectively transfected or might incorporate a little quantity of GFP gene. The transfection rates achieved with the use of MWNT was similar to DNA microinjection (50%)^12^, but was lower than lentiviral vectors (100%)^58^, however, with MWNT nanocarriers was not necessary to manipulate the PZ. The manipulation of PZ of early embryos can impair the development of embryos since the PZ had vital roles in preimplantation embryonic development *in vivo*^59^,^60^. Additionally, all techniques currently available for embryos transfection are quite laborious, because each embryo must be individually micromanipulated which diminish the viability of the embryo.

MWNT is a nanocarrier for transfection of embryo without using equipment for micromanipulation. This reduces the production cost of the embryo transfection for two reasons: firstly, due to the costly equipment, which limits the procedure in many research laboratories. Secondly, due to the necessity of a highly specialized and trained technician to perform the individual embryo micromanipulation. Potentially, the use of MWNTs can be far simpler and less laborious when compared to other techniques, since after its complexation with DNA, its inclusion in the culture medium used for the embryo growth *in vitro* could promote the transfection of multiple simultaneous embryos.

Therefore, the use of the MWNTs as gene delivery into bovine embryos has a promising potential as an alternative method for delivery DNA because of its simplicity and good efficiency.

In present study, for the first time, we showed that MWNTs, could efficiently delivery the GFP gene into embryos, and the exogenous GFP gene was successfully expressed. Therefore, MWNT can be considered as a new carrier for the delivery of DNA into mammalian embryos and may have new good biotechnological applications for this nanomaterial.

In conclusion, this investigation demonstrates for the first time in literature that MWNTs can cross the PZ delivery of pDNA into *in vitro*-fertilized bovine embryos without impairing their ability to hatch or increased embryonic degenerations rates. In addition, despite an increase in the apoptotic index, Real-time PCR assay results showed that MWNT did not alter the gene expression levels in the embryos. Finally, this new biotechnological application of CNT is a much more practical alternative, fast and low cost than current techniques available for gene delivery into embryos. Thus, the use of MWNT as vector to introduce foreing DNA in mammalian embryos could be an attractive alternative to generate transgenic animals.

## Methods

### Synthesis of MWNTs

The MWNTs were synthesized using a floating catalytic chemical vapor deposition process using ferrocene and ethylene as the transition metal and carbon precursors, respectively. After the synthesis, the MWNTs were submitted to a simple purification process by washing and filtering several times with isopropyl alcohol in a Millipore filtration system to remove any non-reacted ferrocene and other carbon impurities. After the cleaning process, the MWNTs were dried at 80 °C for 12 h.

### Thermogravimetric analysis (TGA)

The thermal stability of the MWNTs was determined using a TA Instruments SDT-2960 equipment (TA Instruments) in a dry air atmosphere flow of 100 mL min^−1^ with a heating rate of 5 °C min^−1^. The samples (4.7 ± 1.0 mg) were heated from 25 to 1000 °C^61^. Three samples were used to characterize the material.

### Scanning Electron Microscopy (SEM) and Transmission electron microscopy (TEM)

The carbon materials used in this experiment were characterized by SEM using a Quanta 200 FEI-FEG (FEI Company) microscope at 5 kV and by TEM using a FEI Tecnai G2 Spirit electron microscope at 120 kV.

### Preparation of aqueous MWNTs

MWNTs were dispersed in 2 μg ml^−1^ fetal calf serum (FCS) and treated with ultrasonic agitation under 200 W of power, a 24 kHz working frequency and 50% pulse factors per second (UP200, Hieslcher-Germany) for one minute at 4 °C. Afterwards, MWNTs were diluted in CR2aa medium (final concentration of 0.2 μg ml^−1^) and 10% FCS; they were subsequently used for embryo culture. We used 0.2 μg ml^−1^ of MWNT, according to Munk *et al*.^26^.

### Collection and *in vitro* maturation of oocytes

All applicable guidelines for biosafety were followed, accordingly to procedures approved by the Biosafety Internal Commission, protocol number 03/2012. Bovine oocytes used to *in vitro* embryo production were obtained from discarded ovaries collected at commercial slaughterhouse for meat processing and no live vertebrate animals were involved in the experiments. Follicles with 2–8 mm diameter were aspirated, and oocytes with homogeneous cytoplasm were selected. Maturation was performed in tissue culture medium (TCM-199) supplemented with 20 mg ml^−1^ of follicle stimulant hormone, 0.36 mM sodium pyruvate and 50 mg ml^−1^ streptomycin-penicillin in a humidified atmosphere of 5% CO_2_ at 38.5 °C for 24 h.

### Fertilization and *in vitro* culture

The *in vitro* fertilization was performed with 100 μl drops of Fert-TALP supplemented with 2 × 10^6^ spermatozoa ml^−1^, 20 μg ml^−1^ of heparin and 6 mg ml^−1^ of fatty acid-free BSA fraction V for 21 h in a humidified atmosphere of 5% CO_2_ and 38.8 °C in air. The embryo production was performed according to the methodology described by Camargo *et al*.^62^. On day six post-fertilization embryos at the blastocyst stage were randomly distributed into two culture groups: the control group (without MWNTs; n = 119) and the treated group (with 0.2 μg ml^−1^ MWNTs; n = 131). Embryos in both groups were cultured in CR2aa medium, supplemented with 10% FCS and granulosa cell monolayer for 72 h in microdrops covered by mineral oil and under 5% CO_2_ at 38.5 °C in air and 95% humidity.

### Raman spectroscopy

The MWNT-exposed embryos (n = 8, for 24 h) were washed with phosphate-buffered saline (PBS) to remove adsorbed MWNT in the surface and dehydrated in lamina at room temperature before analysis. Raman spectra were collected at room temperature for the control and MWNT-exposed embryos (Andor^TM^ Technology – shamrock sr-303i). The Raman measurements were performed on an inverted optical microscope (Nikon – Eclipse TE2000-U) in a confocal configuration, with the addition of an x, y-stage for raster-scanning samples. Light from a He-Ne laser (λ = 632.8 nm) was focused onto the surface of the sample using an oil-objective with 60x magnification and a numerical aperture NA = 1.4. Raman scattered light was collected by the same microscope objective used for backscattering and recorded using either a single-photon counting avalanche photodiode (APD – Perkin Elmer Optoelectronics – model: SPCM-AQR-14) for Raman imaging or a spectrograph with an air charged-coupled device (CCD – Andor^TM^ Technology - iDus) for spectral analysis. To study the pDNA interaction with MWNT, spectra were collected on a Bruker RFS 100 equipment excited with a Nd+3/YAG laser operating at 1064 nm, equipped with a InGaAs detector cooled with liquid nitrogen and a spectral resolution of 4 cm^−1^. An average of 512 scans were collected with a laser power of 700 mW directed at the sample.

### Relative mRNA quantification by Real-Time PCR

The relative quantification was performed in triplicate using real-time PCR (ABI Prism 7300 Sequence Detection Systems, CA, USA). The reactions were prepared using a mixture of SYBR^®^ Green PCR Master Mix (Applied Biosystems), primers, nuclease-free water and cDNA. For *PRDX1*, *HSP70.1* and *BAX* genes, 600 ng cDNA was used per reaction; for *GAPDH* gene, 200 ng cDNA was used per reaction. The cDNA template was denatured at 95 °C for 10 min followed by 45 cycles of 95 °C for 15 sec, gene-specific primer annealing temperature for 30 sec (Supplementary Table 1), and elongation at 60 °C for 30 sec. *GAPDH* transcripts were used as the endogenous control, and embryos from the control group were used as calibrators.

### Terminal deoxynucleotidyl transferase uracil nick end labeling (TUNEL) staining

The embryos were stained using a commercially available kit (Dead End Fluorimetric TUNEL System, Corporation Madison, WI, USA) according to the manufacturer’s instructions. Briefly, embryos were fixed in 4% (v/v) paraformaldehyde at 4 °C and then permeabilized with 0.2% (v/v) Triton X-100, both in PBS. After permeabilization, the samples were incubated in 100 μl drops with the terminal deoxynucleotide transferase enzyme and 90% (v/v) staining solution (dUTP fluorescein conjugate) for 1 h at 37 °C in a dark humid chamber. Afterwards, the embryos were stained with Vectashield plus 4′6-diamidino-2-phenylindole (DAPI) and mounted on slides for evaluation by fluorescence microscopy. The total number of cells and number of apoptotic cells (TUNEL+cells) per embryo were counted, and the apoptotic cell index was calculated as the ratio of apoptotic cells/total number of cells.

### Gene-Transfer Study

pDNA-MWNT complexes were prepared by mixing 0.2 μg of pDNA (pLGW) with 0.02, 0.2 or 0.4 MWNT in 20 μl médium CR2aa and in 150 mmol L^−1^ NaCl to obtain different pDNA:MWNT mass ratios (1:1, 1:10 and 1:20). Complexes were incubated for 30 min at RT to allow complete complexation to occur. After 6 h post-fertilization, the embryos were *in vitro* exposed to pDNA only alone and different pDNA-MWNT complex in 50 μl drops in CR2aa serum-free medium. Transfection was allowed to proceed for 12 h at 38.5 °C in an atmosphere of 5% CO_2_. Subsequently, the embryos were transferred to CR2 medium containing 2.5% FCS under 5% O_2_, 5% CO_2_ at 38.5 °C in air and 95% humidity for 72 h.

### Fluorescence Microscopy

GFP expression in the transfected embryos in day 3 (72 h post-fertilization) was observed under fluorescent microscope (Zeiss Germany Axioplan). Microphotographs were taken using a digital camera (Sony, Cyber-Shot). The transfection efficiency was calculated as the percentage of fluorescent embryos out of the total number of embryos.

### Detection of GFP transgene by real time PCR analyses

PCR was performed with transfected and non-transfected embryos using a set of primers specific for GFP that amplify a fragment of 214 bp (Supplementary Table 1). Embryos were rinsed PBS and were transferred to 0.2 ml PCR tubes containing 10 μl of DNA extraction solution (5X PCR buffer and 3 mg/mL proteinase K) and frozen at −20 °C until used. Embryos were incubated for 2 h at 50 °C, then for 10 minutes at 95 °C to inactivate proteinase K before PCR. The amplification was carried out in a PCR; ABI Prism 7300 Sequence Detection Systems. The first step was DNA denaturation at 95 °C for 15 min. The second step consisted of 40 cycles of amplification at the following temperatures: 94 °C for 15 sec for DNA denaturation, 61 °C for 30 sec for primer annealing and a final extension step at 60 °C for 30 sec. After each PCR run, a melting curve analysis was performed to confirm that a single specific product was generated. Negative controls, comprising the PCR reaction mixture without nucleic acids, were also run with each group of samples. The reaction mixture of 25 μl containing 12.5 μl mixture of SYBR Green PCR Master Mix, nuclease-free water and 0.5 μM of each primer was added to the material to be amplified. PCR products were separated in 2% agarose gels, stained with ethidium bromide and examined under UV light. Controls including non-transfected embryos or plasmid template were carried out.

### Statistical analysis

Data for the hatching and degeneration rates after MWNT exposition were analyzed using a Chi-square test. The data corresponding to the total cell number and apoptotic cell index were tested for normality by the Shapiro-Wilki test and then subjected to ANOVA; differences among the means were compared by the Student Newman Keuls (SNK) test using the General Linear Model (GLM) of the SAS Software package version 9.1 (SAS Institute Inc., Cary, NC, USA). p values less than 0.05 were considered significant. The data corresponding to the relative gene expression was analyzed using the Pair Wise Fixed Reallocation Randomization test performed by REST^®^ software^63^. The relative expression values are presented as mean ± SEM.

## Additional Information

**How to cite this article**: Munk, M. *et al*. Efficient delivery of DNA into bovine preimplantation embryos by multiwall carbon nanotubes. *Sci. Rep.* **6**, 33588; doi: 10.1038/srep33588 (2016).

## Supplementary Material

Supplementary Information

## Figures and Tables

**Table 1:** Hatching and degeneration rate in bovine blastocysts cultured with and without MWNTs (0.2 μg ml^−1^).

Exposure period	N	Group	Hatching (%)	Degeneration (%)
24 h	68	Control	89.71	8.82
	71	MWNT	83.10	5.63
48 h	68	Control	91.18	13.24
	71	MWNT	87.32	14.08
72 h	68	Control	92.65	14.71
	71	MWNT	87.32	15.49

There were no differences among groups (Chi-square, p > 0.05).

N = number of embryos.

**Table 2:** Total cell number and apoptotic cell index (mean ± S.E.M.) in bovine blastocysts expanded or hatched cultured without and with MWNTs-exposed for 72 h.

Group	N	Total cell number	Apoptotic cell number	Apoptotic cell index (%)
Control (without MWNT)	21	425.85 ± 45.69^a^	93.53 ± 19.84^a^	19.74 ± 2.83^a^
MWNT (0.2 μg ml^−1^)	30	288.08 ± 36.27^b^	111.64 ± 22.17^a^	33.01 ± 3.72^b^

Means with different superscripts within a column differ at p < 0.05 (ANOVA).

N = number of embryos.
