# Supplementary material for: Efficient delivery of DNA into bovine preimplantation embryos by multiwall carbon nanotubes
Source: Sci Rep. 2016 Sep 19;6:33588. doi: 10.1038/srep33588 (PMC5027538; doi:10.1038/srep33588)
Supplement: Supplementary Information [file srep33588-s1.doc]

**Supplementary Figures and Tables for**

**Efficient delivery of DNA into bovine preimplantation embryos by multiwalled carbon nanotubes**

Michele Munk, Luiz O. Ladeira, Bruno C. Carvalho, Luiz S. A. Camargo, Nádia R. B. Raposo, Raquel V. Serapião, Carolina C. R. Quintão, Saulo R. Silva, Jaqueline S. Soares, Ado Jorio, Humberto M. Brandão

**Supplementary Table**

**Supplementary Table 1**. Primer sequences used for relative gene expression analysis and detection of GFP transgene by real-time PCR.

| Gene name | Primer sequences (5’-3’) | Annealing temperature (°C) | Fragment size (base pair) |
| --- | --- | --- | --- |
| PRDX1 | F-ATGCCAGATGGTCAGTTCAAG R-CCTTGTTTCTTGGGTGTGTTG | 53°C | 224 |
| HSP70.1 | F-AACAAGATCACCATCACCAACG R-TCCTTCTCCGCCAAGGTGTTG | 59°C | 275 |
| BAX | F-TTGCTTCAGGGTTTCATCCAGGA R-CAGCTGCGATCATCCTCTGCAG | 64ºC | 174 |
| GAPDH | F-CCAACGTGTCTGTTGTGGATCTGA R- GAGCTTGACAAAGTGGTCGTTGAG | 53ºC | 237 |
| GFP | F-TGGGCGTGGATAGCGGTTTGAC  R-AAGCCAGTAAGCAGTGGGTTCTCTA | 61°C | 214 |

**Supplementary Figures**

**
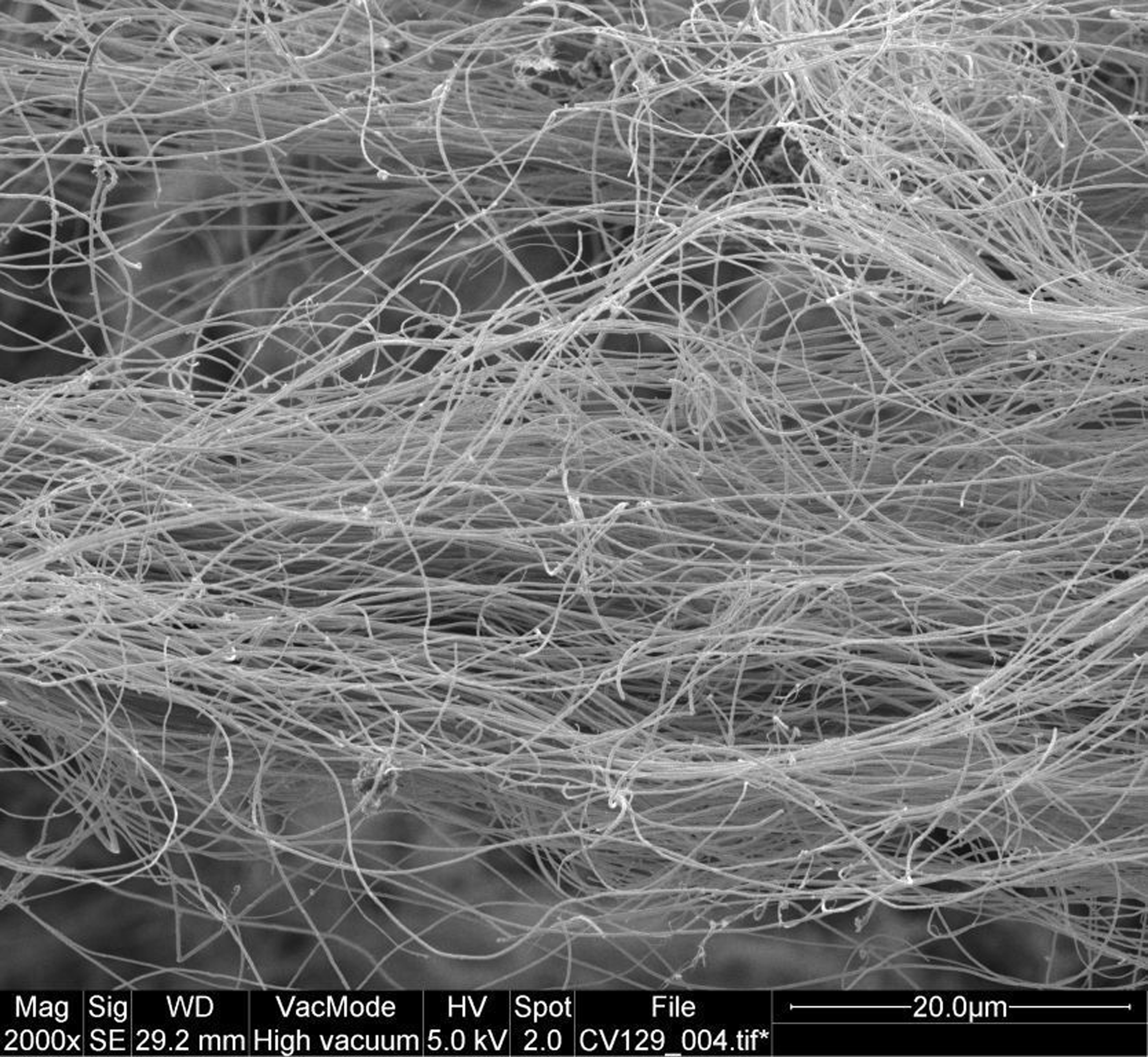
**

**Supplementary Figure 1:** SEM image of the MWNTs used in the experiment.

**
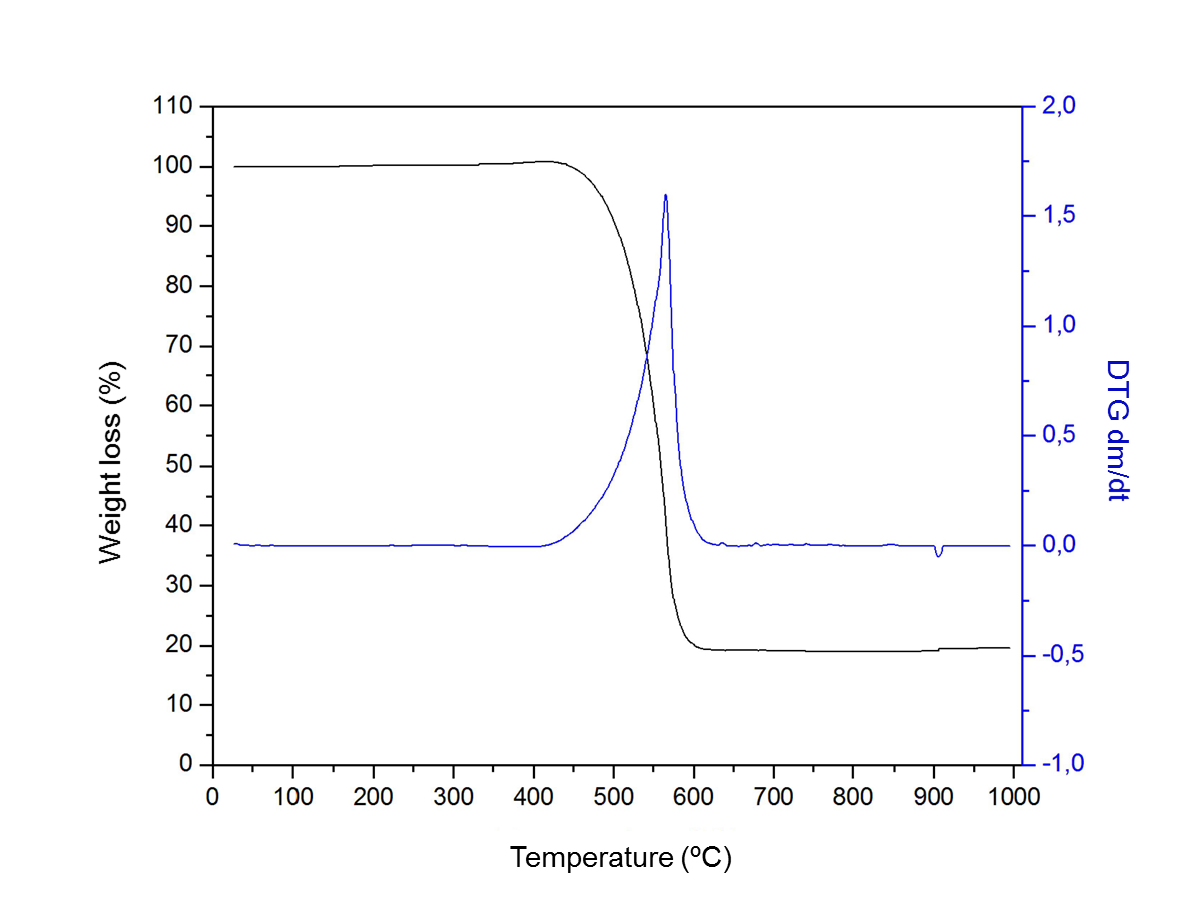
**

**Supplementary Figure 2:** Weight loss versus decomposition temperature by TGA of the MWNTs.


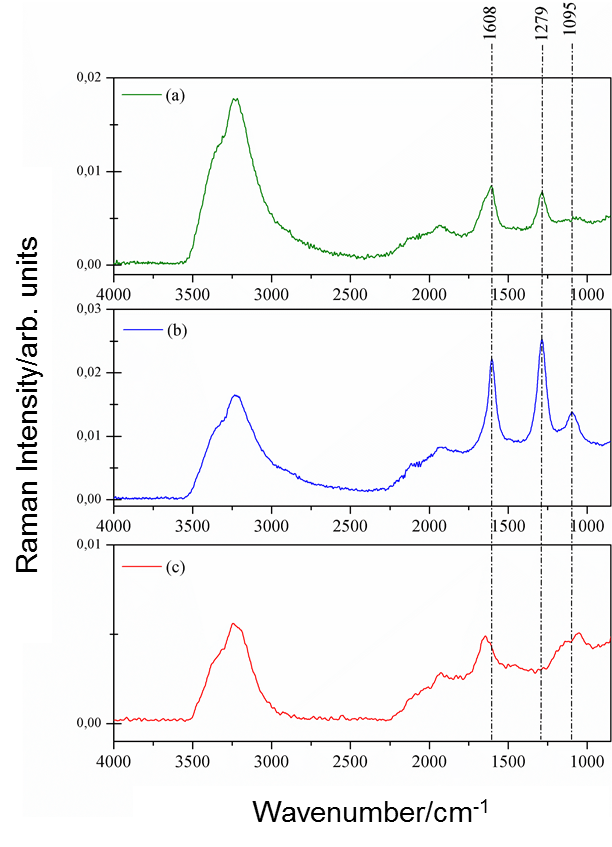


**Supplementary Figure 3:** The Raman spectra of the MWNTs (a), the pDNA-MWNTs complexes (b) and pDNA (c).
